# Design, adaptation and content validation of the Sheffield Profile for Assessment and Referral for Care in Colombian Spanish (SPARC-Sp-Col)

**DOI:** 10.1186/s12904-026-02030-2

**Published:** 2026-02-21

**Authors:** Cindy V. Mendieta, Esther de Vries, Jose A. Calvache, Sam H. Ahmedzai, Gillian Prue, Joanne Reid

**Affiliations:** 1 PhD Program in Clinical Epidemiology, Department of Clinical Epidemiology and Biostatistics, Faculty of Medicine, Pontificia Universidad Javeriana, Bogotá, Colombia; 2 Department of Collective Health Nursing, Faculty of Nursing, Pontificia Universidad Javeriana, Bogotá, Colombia; 3 Department of Nutrition and Biochemistry, Faculty of Science, Pontificia Universidad Javeriana, Bogota, Colombia; 4 Department of Clinical Epidemiology and Biostatistics, Faculty of Medicine, Pontificia Universidad Javeriana, Bogota, Colombia; 5 School of Nursing and Midwifery, Queen’s University Belfast, Belfast, UK; 6 Department of Anesthesiology, Faculty of Health Sciences, Universidad del Cauca, Popayan, Colombia; 7 Department of Anesthesiology, Erasmus University Medical Center Rotterdam, Rotterdam, The Netherlands; 8 Department of Oncology, The University of Sheffield, Sheffield, UK

**Keywords:** SPARC, Holistic needs assessment, Palliative care, Colombia, Validation

## Abstract

**Background:**

Palliative Care (PC) aims to improve the quality of life of individuals with life-threatening illnesses through the early identification and management of holistic needs. Many Colombians in need of PC die without having access to it. When we previously adapted and validated the Sheffield Profile for Assessment and Referral to Care for Spanish-speaking populations (SPARC-Sp), users reported unmet needs and challenges in comprehension and self-administration, particularly among those with lower literacy levels. We designed, culturally adapted, and validated a Colombia-specific module, SPARC-Sp-Col, as an enhancement to SPARC-Sp, to better capture the holistic PC needs of Colombian patients.

**Methods:**

We used a five-step qualitative methodology: (1) preliminary adaptation of a Colombia-specific module as add-on to SPARC-Sp for the Colombian context; (2) online expert panel review; (3) integration of feedback and iterative refinement; (4) cognitive interviews with patients; and (5) focus groups with healthcare professionals, patients, and caregivers from across Colombia.

**Results:**

The iterative adaptation process led to the refinement of item language for improved clarity and cultural resonance, while maintaining semantic equivalence with its original version. We included 17 new items to reinforce existing domains and a dedicated Colombia-specific module to address unmet local needs (5 of them) including housing conditions, violence and navigating the health care system. The evaluation of the resulting tool, SPARC-Sp-Col, demonstrated good content validity according to Aiken’s V (> 0.5), reflective of feedback from expert consensus, allied and social healthcare professionals, patient and caregivers.

**Conclusions:**

SPARC-Sp-Col is a culturally adapted, content-validated instrument that expands SPARC-Sp by incorporating a Colombian-specific module. It enables a more accurate and context-sensitive assessment of the holistic care needs of cancer patients in Colombia. Its development marks a key step toward improving the timely and equitable delivery of PC in a country where access to appropriate support is often lacking.

**Supplementary Information:**

The online version contains supplementary material available at 10.1186/s12904-026-02030-2.

## Background

Palliative Care (PC) aims to improve the quality of life of patients and families with life-threatening illnesses by preventing and alleviating suffering through the early identification and management of holistic needs [1, 2]. Holistic needs assessment (HNA) in PC involves recognising any changes or abnormalities including and beyond the physical and psychological levels that may affect the overall health, general wellbeing or quality of life of an individual and their family. Thus, HNA encompasses the identification of physical, emotional, spiritual, environmental, social, sexual, financial and cultural needs [3].

In Latin America in 2020, an estimated 1,562 PC teams were available. A team is composed of medical and/or nursing staff where at least one member of the team is trained in PC. Extended teams may include psychology, physiotherapy, social work and chaplaincy [4]. PC provision was highest in Uruguay (24.5 teams per million), followed by Costa Rica and Chile [4]. Of these, 75% of the teams were based in hospital settings [4]. Five Latin American countries -Costa Rica, Chile, Mexico, Colombia, and Peru- have established national legislation on PC [4]. In Colombia, according to the Colombian Palliative Care Observatory, 39% of the chronically ill Colombian population could have PC needs and approximately three out of ten people died while waiting for PC [5]. Multiple barriers contribute to the poor access to PC, including limited availability of PC services in some areas, lack of awareness and taboos regarding PC among different stakeholders (policymakers, health professionals, general public), myths, cultural and social barriers framed by beliefs about death, concept of a ‘good death’, and the potential for opioid misuse [6]. In addition, healthcare professionals describe difficulties in conducting HNA early enough, contributing to patients accessing PC very late - at the end of life or in very advanced stages of their disease [7].

To date, only three tools for assessing holistic needs in PC have undergone validation in Colombia [8–11], among which the Sheffield Profile for Assessment and Referral to Care (SPARC) currently has the most comprehensive evaluation of reliability and validity evidence, including linguistic, cross-cultural, content, internal structure, criterion, and construct validity [10, 11]. SPARC is a UK instrument [12] for holistic needs identification validated in Poland [13], Korea [14, 15], Taiwan [16], and in the Colombian context as SPARC-Sp [10, 11]. SPARC-Sp was designed to be a self-administered instrument with eight domains and 56 questions [10, 11]. However, during its initial validation [10, 11] several opportunities were identified for local improvement, given the difficulty in understanding some concepts and the impossibility of self-administration for the relatively large part of the Colombian population with low educational levels [17]. This research aimed to resolve those issues by designing, culturally adapting, and validating the content of the SPARC-Sp-Col instrument for assessing holistic needs in Colombian patients with chronic diseases including cancer from rural and urban areas.

## Methods

### SPARC-Sp instrument

SPARC-Sp is a self-administered instrument with eight domains: communication and information (7 questions); physical symptoms (21 questions); psychological (9 questions); religious and spiritual (2 questions); independence and activity (3 questions); family and social life (4 questions); treatment (2 questions); and personal issues (3 questions) making a total of 56 questions. From the total, four questions are dichotomous (yes/no). The remaining questions have a four-point Likert scale with labels of “not at all” [score of 0], “a little” [score of 1], “quite a bit” [score of 2], and “a lot” [score of 3]” [11]. A score of three is an indication that the patient has a significant PC need [11]. In addition, two open-ended questions are included to probe for additional concerns and worries [11]. Permission for access, use and modification of the SPARC instrument was granted by SHA, its original developer.

**Fig. 1:**
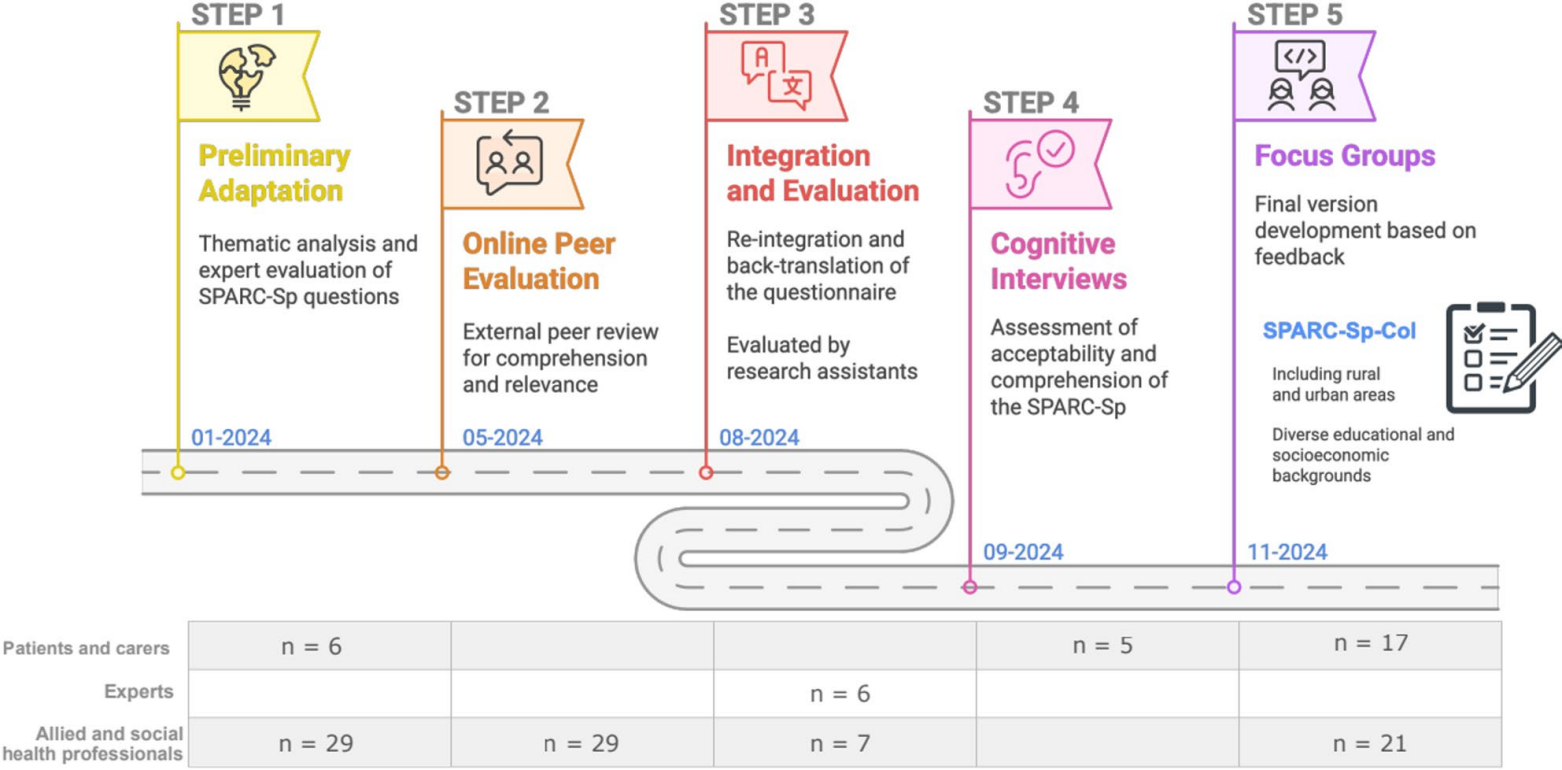
Methodological conduct of this research. Infographics created with Napkin AI [20]

This study was grounded in a social constructivist paradigm, whereby participants actively and iteratively contributed to the co-construction of knowledge, influenced by social and cultural contexts [18]. Fig. 1 illustrates the processes carried out for the methodological conduct of the research presented within this paper, following the guidelines of the Consolidated Criteria for Reporting Qualitative Research (COREQ) (Additional file 1) [19], together with the dates and the number of participants. Each of the 5 steps presented are described in the following sections.

### Step 1: Preliminary adaptation of SPARC-Sp-Col

The first step (January 2024) aimed to identify the preliminary adaptations required to make SPARC-Sp an easy-to-understand instrument for Colombian patients with chronic diseases including cancer and low to moderate literacy skills. SPARC-Sp has a final open-ended question asking patients if they have any holistic care needs that are not covered by the previous questions of the instrument [12]. Based on the findings of the original SPARC-Sp validation of this open-ended question [12], we conducted a thematic analysis to identify the main categories not covered by SPARC-Sp-Col. From this analysis the following were found to be relevant within Colombia but missing from SPARC-Sp: social and geographical barriers; economic and legal issues; health system barriers; occupation; and violence and insecurity [21].

Social and geographical barriers encompassed difficulties in mobility to access health centres; economic and legal issues referred to difficulties in accessing pensions, or the insurmountable challenge of settling financial debts and the requirement of legal procedures; health system barriers included lack of access to health services, referrals and medical appointment requirements; occupation referred to unemployment and difficulty in returning to work; and violence and insecurity comprised armed conflict, food and nutrition insecurity and sexual abuse [21]. A deductive analysis following the steps established by Fife et al., [22] was carried out to identify: (i) modification of words to enhance understanding; (ii) new items that responded to eight domains contemplated in the original version of SPARC-Sp; and (iii) new items that represented unmet needs related to the Colombian context (Colombian Module for SPARC-Sp-Col).

### Step 2: Online peer evaluation

The second step (May 2024) aimed to assess the relevance and understanding of the modifications and additions made to SPARC-Sp (SPARC-Sp-Col). The preliminary prototype of SPARC-Sp-Col was drafted into an online Microsoft Forms file [23]. This online version included the items accompanied by questions about their relevance (Likert scale: 0 – Not relevant to 5- Highly relevant) and comprehensibility (yes/no) of items added (*n* = 18) or reworded (*n* = 15) with the possibility to suggest further changes. The questionnaire was sent via email to potential participants and was returned anonymously.

Potential participants included allied and social healthcare professionals and spiritual advisors from rural and urban areas of Colombia with cancer expertise and who had participated in a previous multidisciplinary research project on PC in Colombia [24]. In June 2024, these potential participants (*N* = 62) were invited through social networks and email, of which 29 agreed to participate (47% response). Previous works suggests that the recommended sample size requirement for this type of study is 15–30 persons [25].

We conducted a thematic analysis on the data obtained from this Microsoft Forms following the methodology described by Fife et al., which encompasses the development of the research question, operationalisation of theory, purposive sampling, coding and analysis, and theorization [26]. We evaluate comprehensibility and relevance and estimated the Aiken V Coefficient to determine the proportion of participants who had a positive evaluation of the proposed SPARC-Sp-Col items [27] using R Studio 4.3.3 [28]. The open-ended questions were analysed through a thematic analysis by CVM, supervised by EdV and JAC [10].

### Step 3: Integration and evaluation

In the third step (August 2024) we evaluated the semantic equivalence of SPARC-Sp-Col (step 2) through the judgement of experts, defined as those with experience with the assessment instrument (SPARC-Sp) [29]. This process involved feedback from bilingual experts and the multidisciplinary team of researchers in charge of the field application of the original version of SPARC-Sp. We also determined the comprehensibility and relevance of new items by researchers with experience in PC in Colombia and the application of SPARC-Sp.

Two bilingual researchers (CVM and EdV), with good fluency in Spanish and English, translated the version resulting from step 2 to English for the native English-speaking researchers to review the changes made (GP, JR and, SHA) following the ISPOR good practice guidelines for the translation and cultural adaptation of patient-reported outcome measures [30]. This translation sought a conceptual, social and cultural equivalence rather than a literal translation, as the English version will not be used in practice [31]. Moreover, some of the new questions for Colombian people would not have resonance in the British health and social care context due to differences in the functioning of the health system and linguistic variations, particularly in the use of colloquial expressions. The translated version was provided in an online Microsoft Forms document where the UK experts (GP, JR and, SHA -original developer of SPARC) [3] evaluated the relevance of the new items (Likert scale: 0 – Not relevant to 5- Highly relevant) and had the possibility to suggest modifications. These suggested modifications were translated into Spanish by CVM under the supervision of EdV and were consolidated in a final report that featured the final version of SPARC-Sp-Col in Spanish and English. Discrepancies were resolved through an online meeting via Microsoft Teams with the Colombian (CVM, EdV, JAC) and UK (GP, JR and, SHA) research team members.

This revised version of SPARC-Sp-Col was presented to four multidisciplinary researchers with experience in the application of original SPARC-Sp in Colombia and three researchers with PC experience to determine the comprehensibility of the items and the relevance of the new additions to the original instrument. Participants completed an anonymous evaluation through an online form that was subject to thematic analysis.

### Step 4: Cognitive interviews

Step 4 (September 2024) aimed to consolidate previous phases of the study through the assessment of acceptability, relevance, comprehensibility and completeness of SPARC-Sp-Col from the patients’ and carers’ perspective. A paper copy of the version of SPARC-Sp-Col produced in step 3 was presented in five cognitive interviews to assess the acceptability, relevance, comprehensibility and completeness of the modifications made and the new items. Eligibility criteria for these cognitive interviews were Colombian adults (> 18 years old), who would be available for an interview of approximately 90 min and who would agree to participate. This sample size was based on the standards for content validity established by COSMIN (COnsensus-based Standards for the selection of health Measurement INnstruments) [32]. Participants (Additional file 2) were selected through purposive sampling [26] with the aim of including diversity of gender, age, geographic location and educational level, from the contact list of participants of the previous project and through the networks of the principal investigators following the eligibility criteria.

The interviews were conducted face-to-face by CVM in private classrooms at the University or at the participants’ homes and were recorded and transcribed verbatim, after which a thematic analysis was carried out [22]. The findings were integrated into a report, translated and back-translated for presentation and discussion with the research team including the original SPARC developer (SHA).

### Step 5: Focus group with discussions

Step 5 (November 2024) aimed to determine the usefulness, difficulties, facilitators and perspectives on the use of SPARC-Sp-Col including the hypothetical needs assessment summary which includes a synthesis of the main needs identified by SPARC-Sp-Col. We conducted four focus groups: two in Bogota, which is the capital city and a very urbanized area; and two in Popayan, which is a small city in a largely rural, relatively poor region. In each area one focus group was conducted with healthcare professionals and allied healthcare professionals and another with patients with chronic diseases and carers of such patients, including cancer patients and carers. The participants in these groups came from both urban and rural areas and represented diversity in perspectives and experiences that enriched the discussions and outcomes of the focus groups. Participants were patients with chronic diseases, carers and allied and social healthcare professionals from rural and urban areas of Colombia. We excluded patients who were hospitalised or unable to communicate due to the methodological conduct of this phase of the research. Purposive sampling was applied, where participants were invited through social networks of the previous project [26]. We planned to include approximately seven people per focus group, seeking diversity in background, gender and age [26].

Each focus group lasted approximately two hours and included a video presentation of SPARC-Sp in Spanish, designed specifically for the group of participants taking part in the activity (patients vs. healthcare professionals) [24]. A printed version of SPARC-Sp-Col was given to each participant. Participants were given the instruction to read SPARC-Sp-Col carefully, and if they so desired, they could complete it. Accompanying the paper version, we presented a fictitious but realistic example of a needs assessment recorded using SPARC-Sp-Col, based on a hypothetical patient (Additional file 3).

Each group was facilitated by a researcher (CVM, EdV, JAC) who oversaw and guided the discussions. During the focus groups, sticky notes were provided so that participants could record their ideas directly onto these, and a participant-moderator from each group was invited to share them and the main findings of their group to the other groups. The interviews were conducted at the university (Bogota) or at a partner institution in the rural area (Popayan).

Focus group were audio-recorded and transcribed verbatim. Analysis was carried out at the culmination of each focus group, with CVM and EdV conducting a thematic deductive analysis following the processes established by Fife et al. [22]. The main categories were discussed, and tiebreakers were resolved by a third researcher (JAC) by consensus. The results were translated into English by a bilingual researcher (CVM) and compiled into a report to a panel of experts in the UK including the original developer of SPARC for further discussion.

## Results

### Step 1: Preliminary adaptation of SPARC-Sp-Col

Based on the observations identified in the previous validation of SPARC-Sp-Col [10, 11], we identified: (i) modification of words to enhance understanding; ii) new items that responded to eight domains of the original version of SPARC-Sp; and (iii) new items that represented unmet needs related to the Colombian context (Colombian Module for SPARC-Sp). We modified 17 items in the domains of communication and information, physical symptoms, psychological, religious and spiritual issues and personal matters of SPARC-Sp (Table 1). Moreover, we identified 10 potential new items that could complement seven of the original eight domains of SPARC-Sp and we created the Colombian module for SPARC-Sp which seven new items related to housing characteristics, armed conflict, lack of a caregiver, difficulty in seeking medical appointments, accessing treatment, medicines or other services, bearing the cost of treatment or medicines, and the requirement to travel to other places to receive care (Table 1).

**Table 1:** Modifications and additions to items during the five steps

Domain	Items		Steps where the item were modified					Type of modification
	Original version	Final version	1	2	3	4	5	
Comunication and information	Other people (please state)	Other persons, services, programmes or support groups (which ones):	X		X			Improve clarity
	* Do you feel that you are not being told the truth about your situation or illness?	Do you feel that you have all the information you need about your health situation or illness?	X	X	X			Use softer and non-judgmental wording
	* Have you been afraid to talk about your health situation or illness?	Have you been afraid to talk about your situation or illness?	X	X				Improve holistic needs assessment
Physical symptoms	Feeling sick (nausea)?	Feeling /sick/ (nausea)?	X					Improve clarity
	Being sick (vomiting)?	Being /sick/ (vomiting)?	X					Improve clarity
	Bowel problems (e.g. constipation, diarrhoea o incontinence)?	Intestinal disturbances (e.g. constipation, diarrhoea, loose or stomach damaged)?	X			X		Improve clarity
	Bladder problems (urinary incontinence)?	Having pain when urinating, bleeding when urinating, itching, urinary incontinence or leaking urine when not in the toilet?	X		X	X		Improve clarity
	Problems sleeping at night?	Having difficulty falling asleep or waking up many times during the night?	X		X			Improve clarity
	Problems with swallowing?	Difficulty swallowing or passing food or drink?				X		Improve clarity
	Being concerned about changes in your appearance?	Because of changes in their physique?	X					Improve clarity
	Feeling that your symptoms are not controlled?	Feeling that your ailments or discomforts have not been controlled?	X					Improve clarity
	*Feeling changes in your vision?	Feeling changes in your vision?	X	X	X			Improve clarity and holistic needs assessment – Small changes in the Spanish wording
	*Feeling changes in your hearing?	Feeling changes in your hearing?	X	X	X			Improve clarity and holistic needs assessment
	* Feeling that you are retaining fluids?	Do you have swelling in your feet or legs?	X	X		X		Improve clarity
Psychological issues	Feeling anxious?	Feeling uneasy, brooding or anxious?	X					Improve clarity
	Feeling confused?	Feeling confused (not understanding what is going on)?	X		X			Improve clarity
	Feeling unable to concéntrate?	Having difficulty concentrating or feeling distracted?	X		X			Improve clarity
	The effect of your condition on your sexual life?	Changes in your sexual life?				X		Improve wording in Spanish
	*Having thoughts that keep you awake at night?	Having thoughts, torments or anxieties that keep you awake at night?	X	X	X	X		Improve clarity
Religious and spiritual issues	Religious or spiritual needs not being met?	Need help on a religious or spiritual level and do not receive it?	X		X			Improving clarity
	*Feel that you need help to make amends or reconcile with someone?	Feeling that you need to make amends or reconcile with someone?	X	X	X	X		Improving holistic needs assessment
Independence and activity	* Losing your independence to make decisions about your life and health situation?	Feeling unable to make decisions about your life or health situation?	X	X	X	X		Use softer and non-judgmental wording
Family and Social issues	*Feeling that your family treats you as if you are not going to get better?	Feeling that your family thinks differently than you do about your health situation or illness?	X	X	X			Use softer and non-judgmental wording
Personal issues	Do you need any help with your personal affairs?	Do you need help with personal matters (paperwork, legal issues, debts, etc.)?	X					Improve clarity
	Financial issues	Money issues	X					Improve clarity
	Other (please state)	Other types of support (education, work, psychology, exercise, therapies, food, communication, etc.).	X		X	X		Improve clarity
	* Do you need help with accessing a disability or retirement pension?	Do you need help to receive a disability pension?	X	X	X			Improve clarity
Colombian module	* Feeling that health staff do not take into account your living situation (transport, condition of the house, money issues, armed conflict, caregivers)?	Feeling that your current living situation or conditions (transport, features of your home - e.g. having to climb stairs, money matters, armed conflict, lack of a caregiver) are not taken into account by health care staff?	X	X		X		Improve clarity
	*Having difficulty in meeting with health professionals (problems with appointments, authorisations, continuity)?	Having difficulty in meeting with health professionals (problems with appointments, authorisations, continuity of care, etc.)?	X	X				Improve clarity
	* Access to treatment or other services?	Be able to demand or follow up on treatment, medicines or other services?	X	X		X		Improve clarity
	* The costs of your treatment or medication?	The price of your treatment or medication?	X	X		X		Improve clarity
	* Displacement to other places for care?	Travel to other places for care?	X	X		X		Use softer and non-judgmental wording and avoid confusion^%^

* New questions added to SPARC-Sp-Col, where the original version corresponds to the first version of each question. ^%^ The word “displacement” in Colombia is usually interpreted as being forcibly displaced because of the armed conflict

### Step 2: Online peer evaluation

The new version of SPARC-Sp that emerged from step 1 (SPARC-Sp-Col) was evaluated by 29 allied and social health professionals working in both rural and urban areas of Colombia (5 general practitioners; 7 medical specialists; 7 nurses; 1 occupational therapist; 1 dietitian; 1 speech therapist, 2 psychologists; 5 spiritual or religious counsellors). Participants were provided with a structured online form where they could rate the acceptability of each of the items and justify the answer behind their choice. The average response time was 40.19 min. Most of the modifications made to SPARC-Sp-Col were widely accepted by the participants (Additional file 4). However, some questions had a lower acceptance due to the difficulty in understanding terms such as ‘appearance’ and ‘anxiety’. Therefore, we identified synonyms or used colloquial terms which were easier to understand.

For the evaluation of the relevance of the new items added to the pre-existing SPARC-Sp-Col domains we applied a Likert scale (5: very relevant to 0: not relevant) which allowed the estimation of the Aiken V coefficient. All new questions, including the Colombian module, were relevant according to Aiken’s V coefficient (> 0.5) (Additional file 5), showing that each question represents adequately the content domain to which it belongs. However, despite the relevance of the last question (Requesting medical appointments), participants felt that it could be implied in other questions in this module, so it was removed.

### Step 3: Integration and evaluation

Four researchers with experience in the application of original SPARC-Sp in Colombia (2 anaesthesiologists, 1 palliative care nurse, 1 rural physicians) and three researchers with PC experience (1 medical epidemiologist, 1 psychologist and 1 nurse) reviewed the modifications and additions to the questions. Based on their feedback, we presented two versions of the 17 questions (the first one kept the original version, and the second one was constructed with the feedback given by the participants). These two options were presented to a panel of seven experts with backgrounds in clinical epidemiology, psychology or PC who selected one of the options as their preferred version and had the option to modify or suggest further adjustments (Additional file 6).

### Step 4: Cognitive interviews

Five cognitive interviews were conducted with participants including people of different ages, backgrounds, occupations, educational levels (Additional file 2). Cognitive interviews were used to assess comprehensibility, difficulty of terms and wording. Feedback from participants identified colloquial terms to exemplify a need (e.g. stomach bug - ‘soltura’), identified synonyms, problems in wording or culturally sensitive terms (e.g. displacement, in Colombia used as a synonym for forced displacement and not a travel to another destination to receive healthcare attention) (Table 2).

**Table 2:** Modifications of the items according to cognitive interviews

Domain	Item	Comments
Physicial symptoms	Bowel problems (e.g.: constipation, diarrhoea or “stomach damage”	The use of “soltura” – “stomach/stomach bug” is suggested as a more colloquial way of giving a description.
	Leaking urine?	We add these terms suggested by the participants as more colloquial ways of exemplifying the construct to be assessed
	Problems with swallowing?	“Swallowing” was a very difficult term for participants to understand, so we used a more colloquial term and added the example of passing food or drink.
	Feeling that you are retaining fluids?	“‘Fluid retention” was not clear to all participants.
Psychological issues	The effect of your condition on your sexual life?	Although the original version was clear, it was not well drafted in Spanish. It certainly did not match with the original instruction of the question
	Having thoughts that keep you awake at night?	Participants suggested more colloquial terms such as “torments’” or “anxieties” are very Colombian ways of explaining that a thought resonates a lot in your head.
Religious and spiritual issues	Feel that you need help to make amends or reconcile with someone?	The wording was changed to improve cohesion with the introduction of the Spanish question.
Independence and activity	Not being able to make decisions about your life or health situation?	The wording was changed to avoid the inclusion of negative words that could cause confusion for the participant.
Personal issues	Other types of support (occupational, psychological, therapeutic, nutrition and food, leisure and free time, recreational activities-recreation and sport)?	Giving examples helped participants to better understand the question. We also had to change words perceived as difficult for participants such as physiotherapy or rehabilitation to exercise and therapies.
Colombian module	Feeling that health staff do not take into account your living situation (transport, condition of the house, money issues, armed conflict, caregivers)?	Some participants found it strange that we asked this question, because they do not consider it the duty of health staff to analyse these factors. However, all perceived it to be a relevant question. For clarity, it was suggested that we clarify what we meant by features of your home and the term caregivers.
	Continuity of your treatment or medication?	Participants suggested the change of “continuity” as it is a very complex word.
	The costs of your treatment or medication?	Participants suggested an even more colloquial way of asking about cost.
	Displacement to other places for care?	The word “displacement” was difficult to understand and often misinterpreted as in Colombia it is often used to refer to forced displacement because of the armed conflict. Participants suggested to us a much simpler and clearer form

### Step 5: Focus group with discussions

We conducted four focus groups in November 2024 with a total of 38 participants including healthcare professionals, caregivers and patients from Bogota and Popayan. Characteristics of the participants are described in Additional file 7. Following the deductive analysis [22], we established some main categories: (i) Extension and application of SPARC-Sp-Col, (ii) Frequency of use and timing of application, (iii) Summary of needs, (iv) Other suggested modifications for SPARC-Sp-Col, (v) Barriers and (vi) Colombian module. The practical recommendations for the implementation and use of SPARC-Sp-Col according to these stakeholders are described in Table 3. The final version of SPARC-Sp-Col can be consulted in the data in the Additional file 8.

**Table 3:** Practical recommendations for the implementation and use of SPARC-Sp-Col according to stakeholders

Characteristics	Professionals	Patients	Caregivers
Extension and application of SPARC-Sp-Col	Consider the extensive length of SPARC-Sp-Col and the potential difficulties in clinical contexts with a high number of patients.	Express that the length does not necessarily constitute a barrier for them to completing it. Acknowledge that it allows a more personalized and individual assessment of needs and care planning.	
	Recommend independent administration, without presence or help from relatives or caregivers	Prefer self-administration or a facilitator for older adults or individuals with limited literacy skills, different to caregiver.	Suggest caregivers answer it together with the patient, given their closeness, knowledge, and ability to support potential functional limitations.
Frequency of use and timing of application	All propose multiple moments or settings for administration: at diagnosis, prior to medical or specialist consultation, and in waiting rooms or inpatient care during follow-up.		
Summary of needs	All recommend sharing the synthesis of needs identified and their priority by SPARC-Sp-Col with healthcare staff, caregivers, or family members.		
Other considerations	Suggest modifications in response options: replacing “quite a bit/bastante” and “very much/mucho” with “mucho” and “muchísimo”; use of color codes (similar to traffic lights) for response options. Recommend the use of SPARC-Sp with the inclusion of the Colombian module (SPARC-Sp-Col). Propose future adaptations to indigenous languages and Braille. Highlight the need to consider the stress experienced by healthcare staff, patients, and caregivers when addressing needs related to structural determinants.		

### Extension and application of SPARC-Sp-Col

SPARC-Sp-Col was perceived by health professionals as a lengthy instrument, possibly tedious to answer. For these reasons, the professionals expressed concerns regarding its applicability in high patient volume clinical settings. The patient’s perspective concurred that the instrument was lengthy but expressed that in their current situation and life condition, time had a different meaning, and that the length would not be an impediment for them to use the tool. They highlighted that the Colombian module allowed for a more comprehensive and patient-centred self-care needs assessment.

*The only disadvantage would be that the consultation could take a little longer*,* especially in clinics where there is a high volume of patients (Male*,* Dentist*,* periodontics specialist*,* Popayan).*

*That’s another thing you learn with time. Dedicating time to oneself (Male*,* Patient*,* Popayan).*

There was a range of views about the application of SPARC-Sp-Col, with practitioners suggesting that it should be applied independently by the patient, without the need for help from their family member or caregiver, to avoid biasing their responses.

*The relative sometimes says: -“no*,* if he is suffering a lot”- and the relative begins to fill out the forms (…) “Yes*,* I have seen that he is in a lot of pain or that he is sad” but perhaps that is not the case (Female*,* Geriatric resident*,* Bogotá).*

However, caregivers felt that it was important for them to carry out SPARC-Sp if it is possible together with the patient because of their close relationship with the patient, knowledge of the patient and the difficulty of completing the instrument by patients with more advanced clinical or greater clinical deterioration.

*Identify who is the patient and the caregiver; usually it is the caregiver who knows all about the patient (Female*,* Caregiver*,* Cauca rural area).*

*We don’t know what condition the patient is in. And maybe the caregiver knows everything about the patient*,* so the caregiver is the one who supports him/her [the patient] in filling out the form (Female*,* Caregiver*,* Cauca rural area).*

From the patients’ perspective, SPARC-Sp-Col was viewed as a very personal instrument, which could help to identify and communicate their own needs. However, patients considered that filling it out may be difficult for certain patients (e.g. older adults, people with low literacy skills) and the support of a facilitator (possibly a family member or caregiver) who can guide its application may be required.

*It can be with a family member or sometimes it can be filled out without help*,* as this tool is a way to let off steam about one’s health and to communicate issues with the family (Female*,* patient and caregiver*,* from Bogotá*,* hospital alliance group).*

*There are people from the villages*,* there are people from the municipalities*,* there are people from everywhere*,* (…) and if someone does not understand*,* there is the person (facilitator) right there*,* ready to explain and help them (Female*,* Patient*,* Popayan).*

### Frequency of use and timing of application

It was suggested that SPARC-Sp-Col could be applied across a range of settings and time points through a patient’s journey. These included: at the time of request for a medical appointment; at diagnosis; prior to the first consultation with the general practitioner or specialist; and in waiting rooms or during hospitalisation.

*I suggest that a link to fill out SPARC will be given when making an appointment (Female*,* Caregiver*,* Cauca Rural Area).*

*In the first consultation*,* it provides us with a baseline of the patient’s situation and provides an idea of which interventions could be good for a patient. Normally*,* in a consultation*,* it is very difficult to be able to cover all the items or needs that a patient may present with and scores positive (…) (Female*,* Doctor*,* geriatrics resident*,* Bogotá).*

*Sometimes patients are a long time in the hospital and don´t have what to do. I think filling out SPARC could even give them something to do to get out of that little routine a bit and at once provide valuable information (Male*,* Physician and Clinical Epidemiologist*,* Bogotá).*

### Summary of needs

Participants suggested the needs identified by SPARC-Sp-Col could be shared in a needs summary with the healthcare staff, caregivers or family members. This synthesis of needs in a graphical format can be represented as an infographic where it is possible to identify the needs in order of prioritisation for addressing them. Patients, caregivers and healthcare staff considered that this summary of needs could optimise communication and identification of needs, prioritisation of their multidisciplinary approach and reduce delays in appropriate service provision.

*Not only are they going to see the psychological needs*,* but with the summary of needs we already know which professional should be following up and accompanying the patient (Female*,* Psychologist*,* Popayán).*

*I think it is a very important tool because the doctor already has good information at a glance*,* and it would be to ask this [summary of needs]. This is what I see as valuable*,* given the time*,* it is very short (according to SPARC-Sp-Col) (Female*,* Patient*,* Valle del Cauca).*

*The oncologist does not tell you about your sexual side (…) In other words*,* you are left with the feeling that you are embarrassed to ask about it and with that trauma (the cancer) (Female*,* Patient*,* Santander)*.

### Other suggested modifications for SPARC-Sp-Col

There was confusion between the options of “quite a bit/bastante” and “very much/mucho”. In the validation phases, we found that depending on the region within Colombia, “bastante” can be considered as either more or less than “mucho”, causing confusion for participants to identify the logic in the order of answer options (which was meant to represent the ascending level of difficulties). As a solution, participants suggested using “mucho” and “muchísimo” instead of “bastante”/”mucho”. “Muchísimo” represents a more colloquial form and denotes greater intensity or severity. In addition, they suggested using colour coding within the answer options following traffic-light logic (green: ‘no worries or preoccupations’, through to red: ‘highest level of worries’).

This idea of colour coding was well received:

*I’m interested*,* I would like to have this little piece of paper myself and say “yes*,* Doctor. Look what happened*,* this came out in red” (Female*,* Patient and caregiver*,* Bogotá).*

To increase the potential use in multiple populations within Colombia, it was suggested to translate and validate Sparc-Sp-Col in indigenous languages such as Misak, Nasa; and also, to have it available in Braille.

### Barriers

Healthcare professionals expressed concern about the type of needs identified in the domains of personal issues (Do you need help to receive a disability pension? ), and in the Colombia module of SPARC-Sp-Col, because many needs corresponded to structural determinants that would prove difficult for health workers to address. Participants considered that for SPARC-Sp-Col to be used in clinical practice, multiple actions would be needed, such as: raising awareness of the instrument; dissemination of the instrument; training of healthcare personnel in its use; and design and explanation of the possible care pathways to address the needs identified by SPARC-Sp-Col.

*We should have*,* let’s say*,* a roadmap of who we are going to ask for help so that they can help us to fulfil what we are saying [address the needs](…) We have to really document and know how far we can go as an institution*,* because otherwise we are going to give the patient hopes that we will not be able to fulfil. (Female*,* Psychologist*,* Popayán).*

### Colombian module

The Colombian module was widely accepted by patients and caregivers as it links economic needs, necessary travel for care and access to retirement pensions. There was one suggestion to adjust the question on having difficulties in access to pension.

*The economic issue for me is I think that this is the majority*,* if I say that there are 90% of fellow patient in chemotherapy who had to give up work*,* they have had to give up everything (Male*,* Patient*,* Popayán).*

*Question about having to travel to other places to receive care: I put in a lot (second highest score)] (…). Currently I have to pay money*,* they charge me 350*,*000 [Colombian pesos] (Female*,* Patient*,* Popayán).*

## Discussion

Our research has shown the development and positive evaluation of an instrument for assessing holistic care needs in Colombian patients with chronic diseases, including cancer, from both rural and urban areas. This was possible due to the strong community involvement in the design and execution of this exercise as the iterative contact with patients, caregivers and allied and social healthcare professionals was needed to fine-tune the adjustments. Our research showed that multiple rounds of development and evaluation were needed to effectively address the issues identified in earlier validation phases. This approach ensured the tool would be useful across Colombia’s diverse populations, both within and outside the universal healthcare system.

From the comments made in the first validation round of SPARC-Sp [10, 11], we learnt that many patients and caregivers have difficulties in accessing PC for physical, spiritual needs. They also mentioned multiple financial and social difficulties which would need to be addressed to effectively access PC, on whatever level of provision [10, 11, 33]. Addressing these observations led to the development of the “Colombian module” as an add-on to SPARC-Sp. Our validation suggests that the Colombian module enhances the tool’s cultural and contextual relevance by effectively addressing the identification of holistic needs specific to the Colombian population. This adaptation is expected to improve the instrument’s acceptability, comprehension, and usability, particularly among individuals with diverse sociocultural and educational backgrounds.

The multiple small but important linguistic adaptations also show the importance of local testing and adjustment of wording of instruments trying to maintain semantic equivalence [31]. In this study, we identified differences in the understanding of the SPARC-Sp response options (*quite a bit* – “bastante” and *very much* – “mucho”). These cultural nuances in the use of adverbs of quantity have previously been noted in translations from other languages into Spanish and may lead to inadequate linguistic transfers and induce confusion for the reader [34]. The suggestion of traffic light-logic colouring within the Likert scale may help improve this situation.

Other adaptations of SPARC, such as the Korean version, reported challenges in translation and highlighted the need to soften the wording of certain questions and to employ more colloquial language [14]. Similarly, the Taiwanese adaptation indicated that, although the terms were not particularly difficult to understand, there were similarities between some items, difficulties in distinguishing between the roles of hospital and community nurses, and a strong emotional connotation associated with the word “illnesses” [16].

The previous translations and adaptations were completed in settings that, although linguistically and culturally quite different from the UK, were high-income settings and were performed with a relatively highly educated patient populations [14–16]. We conducted the SPARC-Sp-Col study in a middle-income country, with part of the work taking place in a particularly impoverished region that has long been, and continues to be, affected by violent conflict.

Spanish is a language spoken by most of South and Central American populations, but expressions may be differently understood, even between regions of one country [35]. Whereas it is very common to widely use a tool that was translated and validated in one Spanish speaking country [35], this is a convenience that should not be promoted [36], although sometimes for practical reasons it is impossible to avoid. The understanding and acceptability of items vary by region, culture and educational level, and some formulations may really be misunderstood in some regions generating a cultural bias [36]. Although we employed purposive sampling, seeking heterogeneity of the participants, we recognise that in this project we failed to reflect all existing multidiversity within Colombia. However, we believe that, by working with geographically, culturally and socioeconomically very diverse populations, we have managed to reflect and incorporate many of these variations in the development of the current instrument. In addition, the Popayan area was and remains a relatively poor area of the country that suffers from the armed conflict that persists in multiple parts of Colombia, and which creates its specifics needs and barriers for patients [37]. In turn, the inclusion of Bogotá as the capital of Colombia represents an urbanised region, with relevant problems for the guarantee of access to PC, such as migration, forced intra-urban displacement, economic instability, among other determinants [37, 38]. Future research is needed to evaluate how SPARC-Sp-Col performs in different regions of the country.

An important point of discussion in all groups was whether or not the SPARC-Sp-Col should and could be a self-administration instrument, as it was designed to be. Participants agreed that the design of the questions in principle, allowed for self-administration, but only if people are physically and mentally able. There were two main opinions on self-application: caregivers often thought it was important for them to help fill out the instrument, as they believed that they knew the patient well. While some patients agreed this could work well, others said they would answer differently when their caregiver was assisting them– an important argument for strict self-application when possible. Healthcare professionals generally expressed that in practice, caregivers are often very heavily involved and will tend to “help out” filling the form or even take over the complete task of answering SPARC-Sp-Col – highlighting the importance of clear instructions on how, when, where and with whom to apply the instrument.

From the health professionals’ perspective, the identification of PC needs is also influenced by cultural and religious factors, regional difficulties, misinterpretations of PC, age and illness [39]. Other determinants of suboptimal needs identification may stem from the organization of the health system, allocation of resources, PC training, and workload demands [38], all of which contribute to distress among healthcare professionals [40], and may hinder the appropriate administration of SPARC-Sp-Col and the effective addressing of the identified needs.

Regarding caregivers, previous research highlights the role of caregivers in shared decision-making in setting up a care plan [41]. However, it is estimated that about 55% of caregivers of adults with severe chronic illness have never heard of PC, and 40% associate it only with end of life without considering earlier implementation [41].

At the national level, the 2022–2026 Action Plan (Building a Positive Environment for PC in Colombia) is currently underway, urging the strengthening of PC programmes to ensure the continuous care of individuals with palliative needs and their families [42]. Within this framework, SPARC-Sp-Col could be integrated as a tool to support communication and the identification of needs. Moreover, previous prioritisation exercises for the implementation of PC research in Colombia highlight the importance of exploring the needs of the patient as well as their caregivers as an approach to a more comprehensive assessment [43].

## Conclusion

SPARC-Sp-Col is a culturally adapted, content-validated instrument that expands SPARC-Sp by incorporating semantic changes to the wording and a Colombian-specific module. This tool enables a more accurate and context-sensitive assessment of the holistic care needs of cancer patients in Colombia. Its development marks a key step toward improving the timely and equitable delivery of palliative care in a country where structural, cultural, and educational barriers often delay or prevent access to appropriate support. The findings also highlight the importance of validating and if necessary, adapting instruments to the local population and circumstances – even if they speak the same language. This calls for a more comprehensive approach, which could also encompass large indigenous languages as Nasa, Misak, Wayúu and Emberá and the Braille system.

## Supplementary Information

Supplementary Material 1.

## Data Availability

Data are available in anonymised form upon reasonable request to the authors.

## Abbreviations

COREQ: Consolidated Criteria for Reporting Qualitative Research
COSMIN: COnsensus-based Standards for the selection of health Measurement INnstruments
ISPOR: International Society For Pharmacoeconomics And Outcomes Research
SPARC: Sheffield Profile for Assessment and Referral for Care
SPARC-Sp-Col: Sheffield Profile for Assessment and Referral for Care in Colombian Spanish
PC: Palliative Care
HNA: Holistic needs assessment
