# Supplementary material for: Design, adaptation and content validation of the Sheffield Profile for Assessment and Referral for Care in Colombian Spanish (SPARC-Sp-Col)
Source: BMC Palliat Care. 2026 Feb 21;25:68. doi: 10.1186/s12904-026-02030-2 (PMC13001305; doi:10.1186/s12904-026-02030-2)
Supplement: Supplementary file 1 — Supplementary Material 1. [file 12904_2026_2030_MOESM1_ESM.docx]

**Additional file**

**Additional file 1. Consolidated criteria for reporting qualitative studies (COREQ): 32-item checklist**

| **No. Item** | **Guide questions/description** | **Reported on Page #** |
| --- | --- | --- |
| **Domain 1: Research team and reﬂexivity** | | |
| *Personal Characteristics* |  |  |
| 1. Inter viewer/facilitator | Which author/s conducted the interview or focus group? | Page 8 - 9 |
| 2. Credentials | What were the researcher’s credentials? E.g. PhD, MD | Page 1 |
| 3. Occupation | What was their occupation at the time of the study? | Page 1 |
| 4. Gender | Was the researcher male or female? | Page 1 |
| 5. Experience and training | What experience or training did the researcher have? | Page 1 |
| ***Relationship with participants*** | | |
| 6. Relationship established | Was a relationship established prior to study commencement? | Page 6 - 7 |
| 7. Participant knowledge of the interviewer | What did the participants know about the researcher? e.g. personal goals, reasons for doing the research | Page 9 |
| 8. Interviewer characteristics | What characteristics were reported about the inter viewer/facilitator? e.g. Bias, assumptions, reasons and interests in the research topic | Page 6 - 9 |

| **Domain 2: study design** | | |
| --- | --- | --- |
| ***Theoretical framework*** | | |
| 9. Methodological orientation and Theory | What methodological orientation was stated to underpin the study? e.g. grounded theory, discourse analysis, ethnography, phenomenology, content analysis | Page 4 |
| ***Participant selection*** | | |
| 10. Sampling | How were participants selected? e.g. purposive, convenience, consecutive, snowball | Page 6, 8 |
| 11. Method of approach | How were participants approached? e.g. face-to-face, telephone, mail, email | Page 6, 8, 9. |
| 12. Sample size | How many participants were in the study? | Page 8,9 |
| 13. Non-participation | How many people refused to participate or dropped out? Reasons? | Page 6 |
| ***Setting*** | | |
| 14. Setting of data collection | Where was the data collected? e.g. home, clinic, workplace | Page 6, 8,9 |
| 15. Presence of non-participants | Was anyone else present besides the participants and researchers? | Not applicable |
| 16. Description of sample | What are the important characteristics of the sample? e.g. demographic data, date | Additional file 1, 6 |
| ***Data collection*** | | |
| 17. Interview guide | Were questions, prompts, guides provided by the authors? Was it pilot tested? | Page 6 – Additional file 2 |
| 18. Repeat interviews | Were repeat inter views carried out? If yes, how many? | Not applicable |
| 19. Audio/visual recording | Did the research use audio or visual recording to collect the data? | Page 6 - 9 |
| 20. Field notes | Were ﬁeld notes made during and/or after the interview or focus group? | Page 9 |
| 21. Duration | What was the duration of the interviews or focus group? | Page 8 - 9 |
| 22. Data saturation | Was data saturation discussed? | Page 8 |
| 23. Transcripts returned | Were transcripts returned to participants for comment and/or correction? | Page 9 |
| **Domain 3: analysis and ﬁndings** | | |
| ***Data analysis*** | | |
| 24. Number of data coders | How many data coders coded the data? | Page 5, 6, 8, 9 |
| 25. Description of the coding tree | Did authors provide a description of the coding tree? | Page 10 - 15 |
| 26. Derivation of themes | Were themes identiﬁed in advance or derived from the data? | Page 10 - 15 |
| 27. Software | What software, if applicable, was used to manage the data? | Page 6, 7 |
| 28. Participant checking | Did participants provide feedback on the ﬁndings? | Page 9 |
| *Reporting* | | |
| 29. Quotations presented | Were participant quotations presented to illustrate the themes/ﬁndings? Was each quotation identiﬁed? e.g. participant number | Page 20 - 25 |
| 30. Data and ﬁndings consistent | Was there consistency between the data presented and the ﬁndings? | Page 10 - 25 |
| 31. Clarity of major themes | Were major themes clearly presented in the ﬁndings? | Page 10 - 25 |
| 32. Clarity of minor themes | Is there a description of diverse cases or discussion of minor themes? | Page 10 - 25 |

**Additional file 2. Clinical and demographic characteristics**

| **ID** | **Sex** | **Age (years)** | **Geographical setting** | **Occupation** | **Education level** | **Diagnosis** |
| --- | --- | --- | --- | --- | --- | --- |
| 1 | Female | 51 | Cauca | General service staff | Complete primary | Gastrointestinal tract disease |
| 2 | Female | 62 | Boyaca | Laboratory assistant - Retired | Technologist | Hypertension |
| 3 | Female | 53 | Cordoba | General service staff | Incomplete bachelor's degree | Pemphigus vulgaris |
| 4 | Man | 29 | Bogota | Engineer | Master's degree | None |
| 5 | Man | 27 | Bogota | Commercial adviser | Bachelor's degree | Asthma |

**Additional file 3. Hypothetical Case**

- **Case 1:**

Mr. Pedro is 82 years old and does not present any illness. When applying SPARC-Sp-Col no needs are identified (score: 0 for each item). However, six months later, Mr. Pedro reports pain (score: 1 - a little bit), memory loss (score 3: very much). Thus, the summary of needs shows:

- Urgent need (score 3): Memory loss
- Problematic need (score 1): None
- Maintain in routine review (score 0): Pain
- **Case 2:**

Mrs. Joanna is 30 years old, she lives with multiple sclerosis and reports not being worried about not being able to walk. However, she is very worried about losing her independence (score: 3 - very much) and not being able to bathe herself (score 2: quite a bit). The SPARC-Sp-Col application in the domain of independence and activity identified:

- Losing their independence? - Very much (Score 3)
- Changes in your ability to carry out daily activities such as toileting, bathing or going to the toilet) – Quite a bit (Score 2).

In this way the summary of needs shows:

- Urgent need (Score 3): Memory loss
- Problematic need (score 1): None

**Additional file 4. Acceptance of the final version of SPARC-Sp-Col**

| **Final version** | **% of participants who considered the adaptation clear % (N: 29)** |
| --- | --- |
| Other persons, services, programmes or support groups (which ones): | 66 (19) |
| Feeling /sick/ (nausea)? | 83 (24) |
| Being /sick/ (vomiting)? | 79 (23) |
| Intestinal disturbances (e.g. constipation, diarrhoea, loose or stomach damaged)? | 72 (21) |
| Having pain when urinating, bleeding when urinating, itching, urinary incontinence or leaking urine when not in the toilet)? | 79 (23) |
| Having difficulty falling asleep or waking up many times during the night? | 83 (24) |
| Because of changes in their physique? | 55 (16) |
| Feeling that your ailments or discomforts have not been controlled? | 83 (24) |
| Feeling uneasy, brooding or anxious? | 62 (18) |
| Feeling confused (not understanding what is going on)? | 72 (21) |
| Having difficulty concentrating or feeling distracted? | 72 (21) |
| Need help on a religious or spiritual level and do not receive it? | 90 (26) |
| Do you need help with personal matters (paperwork, legal issues, debts, etc.)? | 90 (26) |
| Other types of support (education, work, psychology, exercise, therapies, nutrition, communication, among others). | 83 (24) |
| Money issues | 83 (24) |

**Additional file 5. Relevance of the preliminary modifications of SPARC-Sp-Col**

| **Domain** | **Question** | **Relevance N (%) = 29** | | | | | | **Aiken’s V (CI 95 %)** |
| --- | --- | --- | --- | --- | --- | --- | --- | --- |
|  |  | **5 (Very relevant)** | **4** | **3** | **2** | **1** | **0 (No relevant)** |  |
| Communication and information | Have you been afraid to talk about your situation or illness? | 23 (79.3) | 3 (10.3) | 2 (6.9) | 1 (3.4) |  |  | 0.93 (0.84 – 1.00) |
|  | Do you feel that you have all the information you need about your health situation or illness? | 18 (62.1) | 7 (24.1) | 2 (6.9) | 2 (6.9) |  |  | 0.88 (0.77 – 1.00) |
| Physical symptoms | Feeling changes in your vision? | 15 (51.7) | 6 (20.7) | 1 (3.4) | 3 (10.3) | 2 (6.9) |  | 0.81 (0.67 – 0.96) |
|  | Feeling changes in your hearing? | 14 (48.3) | 6 (20.7) | 1 (3.4) | 4 (13.8) |  |  | 0.84 (0.70 -0.98) |
|  | Do you feel that you are retaining fluids? | 16 (55.2) | 8 (27.6) | 3 (10.3) | 1 (3.4) |  |  | 0.88 (0.76 – 1.00) |
| Psychological issues | Thoughts that keep you awake at night? | 21 (72.4) | 4 (13.8) | 3 (10.3) | 1 (3.4) |  |  | 0.91 (0.81 – 1.00) |
| Religious and spiritual issues | Need help on a religious or spiritual level? | 17 (58.6) | 2 (6.9) | 4 (13.8) | 1 (3.4) | 3 (10.3) | 2 (6.9) | 0.76 (0.60 – 0.91) |
| Independence and activity | Losing your independence to make decisions about your life and health situation? | 20 (69) | 4 (13.8) |  | 2 (6.9) |  | 3 (10.3) | 0.83 (0.69 -0.97) |
| Family and Social Issues | Feeling that your family treats you as if you are not going to get better? | 19 (65.5) | 4 (13.8) | 2 (6.9) | 3 (10.3) | 1 (3.4) |  | 0.86 (0.73 – 0.98) |
|  | Changes to fulfil your role in the family? | 18 (62.1) | 5 (17.2) | 1 (3.4) |  | 2 (6.9) | 3 (10.3) | 0.79 (0.65 – 0.94) |
| Personal issues | Do you need help with accessing a disability or retirement pension? | 19 (65.5) | 6 (20.7) | 2 (6.9) | 2 (6.9) |  |  | 0.88 (0.76 – 1.00) |
| Colombian Module | Feeling that health staff do not take into account your living situation (transport, condition of the house, money issues, armed conflict, caregivers)? | 21 (74) | 3 (11) | 2 (4) | 1(4) | 2 (7) |  | 0.88 (0.76 – 1.00) |
|  | Having difficulty in meeting with health professionals (problems with appointments, authorisations, continuity of care, etc.)? | 23 (78) | 4 (15) |  |  | 2 (7) |  | 0.91 (0.81 – 1.00) |
|  | Access to treatment or other services? | 27 (93) | 1 (4) |  |  | 1 (4) |  | 0.97 (0.90 – 1.00) |
|  | The costs of your treatment or medication? | 23 (78) | 4 (15) | 1 (4) |  | 1 (4) |  | 0.93 (0.84 – 1.00) |
|  | Continuity of your treatment or medication? | 26 (89) | 1 (4) | 1 (4) |  | 1 (4) |  | 0.95 (0.87 – 1.00) |
|  | Travel to other places for care? | 26 (89) | 2 (7) |  | 1 (4) |  |  | 0.97 (0.90 – 1.00) |
|  | Requesting medical appointments? | 23 (78) | 3 (11) | 1 (4) | 1 (4) | 1 (4) |  | 0.92 (0.82 – 1.00) |

**Additional file 6. Voting of each item according to SPARC-Sp research assistants**

| **SPARC Domain** | **Original questions of SPARC** | **Versions** | **Positive voting N (%): 7** |
| --- | --- | --- | --- |
| **Communication and information** | Other people or services | Old*: Other persons or services (please state): | 2 (29) |
|  |  | New: Other persons, services, programmes or support groups (which ones): | 5 (71) |
|  | Do you feel that you have all the information you need about your health situation or illness? | Old*: Do you feel that you are not being told the truth about your situation or illness? | 3 (43) |
|  |  | New: Do you feel that you are not being told the whole truth about your illness or health situation? | 3 (43) |
|  |  | None of the above | 1 (14) |
| **Physical symptoms** | Leaking urine? | Old*: Urine leakage? | 1 (14) |
|  |  | New: Leaking urine when not in the toilet? | 5 (71) |
|  |  | None of the above | 1 (14) |
|  | Not being able to sleep at night? | Old*: Not being able to sleep at night? | 1 (14) |
|  |  | New: Not being able to sleep at night (waking up many times during the night)? | 4 (57) |
|  |  | None of the above | 2(29) |
|  | Feeling changes in your vision? | Old*: Feeling changes in your vision? | 2 (29) |
|  |  | New: Feeling that you can't see well? | 5 (71) |
|  | Feeling changes in your hearing? | Old*: Feeling changes in your hearing? | 2 (29) |
|  |  | New: Feeling that you can't hear well? | 5 (71) |
| **Psychological issues** | Feeling anxious? | Old*: Feeling uneasy? | 1 (14) |
|  |  | New: Feeling uneasy, brooding or anxious? | 6 (86) |
|  | Feeling unable to concentrate? | Old*: Having difficulty paying attention? | 1 (14) |
|  |  | New: Feeling distracted? | 5 (71) |
|  |  | None of the above | 1 (14) |
|  | Thoughts that keep you awake at night? | Old*: Thoughts that keep you awake at night? | 4 (57) |
|  |  | New: Having thoughts that keep you awake at night? | 3 (43) |
| **Religious and spiritual issues** | Need help on a religious or spiritual level? | Old*: Need help on a religious or spiritual level? | 5 (71) |
|  |  | New: No religious or spiritual help? | 1 (14) |
|  |  | None of the above | 1 (14) |
|  | Feeling that you need help to make peace? | Old*: Feeling that you need help to make peace? | 0 |
|  |  | New: Feeling that you need help to make amends or reconcile with someone (family, friends, health staff)? | 7 (100) |
| **Independence and activity** | Losing your independence to make decisions about your life and health situation? | Old*: Losing your autonomy to make decisions about your life and health situation? | 1 (14) |
|  |  | New: Not being able to make decisions about your life or health situation? | 6 (86) |
| **Family and Social issues** | Feeling that your family treats you as if you are not going to get better? | Old*: Feeling that your family treats you as if you are not going to get better? | 3 (43) |
|  |  | New: The way your family treats you? | 2 (29) |
|  |  | None of the above | 2 (29) |
|  | Changes to fulfil your role in the family? | Old*: Changes to fulfil your role with the family? | 3 (43) |
|  |  | New: Not being able to do the things you used to do with your family? | 4 (57) |
| **Personal issues** | Other types of support (occupational, psychological, therapeutic, nutrition and food, leisure and free time, recreational activities-recreation and sport)? | Old*: Other types of support (work, psychology, physiotherapy, food, etc.) | 1 (14) |
|  |  | New: Other types of support (education, work, psychology, rehabilitation, physiotherapy, nutrition, communication, among others). | 6 (86) |
|  | Do you need help with accessing a disability or retirement pension? | Old*: Do you need help with access to disability or retirement pensions? | 0 |
|  |  | New: Do you need help with access to disability, invalidity or retirement pensions due to your illness? | 7 (100) |
|  | Access to treatment or other services? | Old*: Access to treatment or other services? | 0 |
|  |  | New: Access to or continuity of treatment, medicines or other services? | 7 (100) |

* Old version from the Step 2.

**Additional file 7. Demographic characteristics of focus group participants**

| **City** | **Health professionals (N)** | **Professional profiles** | **Patients and caregivers (N)** | **Gender distribution** |
| --- | --- | --- | --- | --- |
| **Bogotá** | **6** | Nursing assistants (n:3), medical epidemiologist (n:1), occupational therapist (n:1), geriatric resident doctor (n:1). | **12** | Men (n:2; 17 %), women (n:10; 83 %) |
| **Popayán** | **11** | Oncology nursing (n:1), auxiliary nursing (n:1), nursing (n:1), paediatric doctor (n:2), psychology (n:1), dentistry (n:1), psychiatric doctor (n:1), rheumatology doctor (n:1), pain and palliative care nursing (n:1) and paediatric intensivist (n:1). | **9** | Men (n: 2; 22%), women (n: 7; 78%) |

**Additional file 8. SPARC-Sp-Col**
